# The LIM-Homeodomain Protein Islet Dictates Motor Neuron Electrical Properties by Regulating K^+^ Channel Expression

**DOI:** 10.1016/j.neuron.2012.06.015

**Published:** 2012-08-23

**Authors:** Verena Wolfram, Tony D. Southall, Andrea H. Brand, Richard A. Baines

**Affiliations:** 1 Faculty of Life Sciences, University of Manchester, Manchester M13 9PT, UK; 2 The Gurdon Institute and Department of Physiology, Development and Neuroscience, University of Cambridge, Tennis Court Road, Cambridge CB2 1QN, UK

## Abstract

Neuron electrical properties are critical to function and generally subtype specific, as are patterns of axonal and dendritic projections. Specification of motoneuron morphology and axon pathfinding has been studied extensively, implicating the combinatorial action of Lim-homeodomain transcription factors. However, the specification of electrical properties is not understood. Here, we address the key issues of whether the same transcription factors that specify morphology also determine subtype specific electrical properties. We show that *Drosophila* motoneuron subtypes express different K^+^ currents and that these are regulated by the conserved Lim-homeodomain transcription factor Islet. Specifically, Islet is sufficient to repress a Shaker-mediated A-type K^+^ current, most likely due to a direct transcriptional effect. A reduction in Shaker increases the frequency of action potential firing. Our results demonstrate the deterministic role of Islet on the excitability patterns characteristic of motoneuron subtypes.

## Highlights

► Embryonic motoneurons show subtype-specific electrical properties ► Common developmental mechanisms predetermine morphology and electrical properties ► Islet suppresses A-type K^+^ channel expression in ventral motoneurons ► Absence of the A-type K^+^ channel increases action potential firing frequency

## Teaser

Wolfram et al. report that early expression of the Lim-homeodomain transcription factor Islet represses the Shaker-dependent fast potassium channel in developing motoneurons. This is indicative that subtype-specific electrical properties in developing neurons are set by intrinsic mechanisms.

## Introduction

Diversity in neuronal signaling is critical for emergence of appropriate behavior. This diversity is reflected in dendrite morphology, axon pathfinding, choice of synaptic partners, transmitter phenotype, and cocktail of ion channels expressed by individual neurons. Many aspects of vertebrate (e.g., chick, zebrafish, and mouse) motoneuron development, including cell specification, axonal pathfinding, and neurotransmitter choice are regulated through expression of LIM-homeodomain transcription factors, including Islet1/2, Lim1/3, and Hb9 (Appel et al., 1995; Hutchinson et al., 2007; Pfaff et al., 1996; Segawa et al., 2001; Song et al., 2009; Thaler et al., 2004). Homologous proteins, and additional homeodomain (HD) proteins such as Even-skipped (Eve), serve similar functions in invertebrate motoneurons (e.g., *C. elegans* and *Drosophila*) (Certel and Thor, 2004; Esmaeili et al., 2002; Fujioka et al., 2003; Landgraf et al., 1999; Landgraf and Thor, 2006; Odden et al., 2002; Thor and Thomas, 1997, 2002). However, the extent to which neuronal electrical properties are similarly predetermined as part of cell-intrinsic developmental mechanisms remains unknown.

Neurons grown in culture often express their normal complement of both voltage- and ligand-gated ion channels (O'Dowd et al., 1988; Ribera and Spitzer, 1990; Spitzer, 1994). This suggests a significant degree of cell autonomy in the determination of electrical properties that presumably facilitates initial network formation. Once part of a circuit, however, such neurons become exposed to synaptic activity. As a result, predetermined electrical properties are modified by a variety of well-described mechanisms (Davis and Bezprozvanny, 2001; Spitzer et al., 2002). Such tuning ensures consistency of network output in response to potentially destabilizing activity resulting from Hebbian-based synaptic plasticity (Turrigiano and Nelson, 2004). The formation of functional neural circuits would seem, therefore, critically reliant on both intrinsic predetermination and subsequent extrinsic activity-dependent mechanisms to shape neuronal electrical properties. Key to understanding how intrinsic and extrinsic mechanisms are integrated will be the identification of factors that regulate predetermination.

The fruitfly, *Drosophila*, has been central to studies that have identified intrinsic determinants of neuronal morphology. Within the *Drosophila* central nervous system (CNS) the transcription factor Islet is expressed in the RP1, RP3, RP4, and RP5 motoneurons (termed ventral motoneurons, vMNs) that project to ventral muscles (Broihier and Skeath, 2002; Landgraf and Thor, 2006; Thor et al., 1999). By contrast, motoneurons projecting to dorsal muscles (e.g., aCC and RP2, termed dorsal motoneurons, dMNs) express a different homeodomain transcription factor, Even-skipped (Eve) (Broihier and Skeath, 2002; Landgraf et al., 1999). Misregulation of these transcription factors is sufficient to alter subtype-specific axonal projections (Broihier and Skeath, 2002; Landgraf et al., 1999). Thus, Eve and Islet constitute what might be considered a bimodal switch with each being deterministic for either dorsal or ventral-projecting motor axon trajectories, respectively.

Here, we report that the presence of Islet is also deterministic for expression of Shaker (Sh)-mediated outward A-type K^+^ current. The vMN and dMN subgroups differ in magnitude of outward K^+^ currents recorded by whole-cell patch clamp. We show that this difference is maintained by endogenous expression of *islet* in the vMNs. We also show that Islet is sufficient to repress expression of a Sh-mediated K^+^ current. By contrast, dMNs, which do not express *islet*, exhibit a robust Sh-mediated K^+^ current. The deterministic function of Islet is evidenced first by the fact that loss of function results in a transformation of total outward K^+^ current in the vMNs to mirror that present in dMNs. Second, ectopic expression of *islet* in dMNs or body wall muscle is sufficient to repress expression of the endogenous Sh-mediated K^+^ current. Thus, in addition to being sufficient to predetermine aspects of neuronal connectivity, Islet is sufficient to specify electrical properties in those neurons in which it is expressed.

## Results

### Dorsal and Ventral Motoneuron Subgroups Show Specific K^+^ Current Profiles

**Figure 1:**
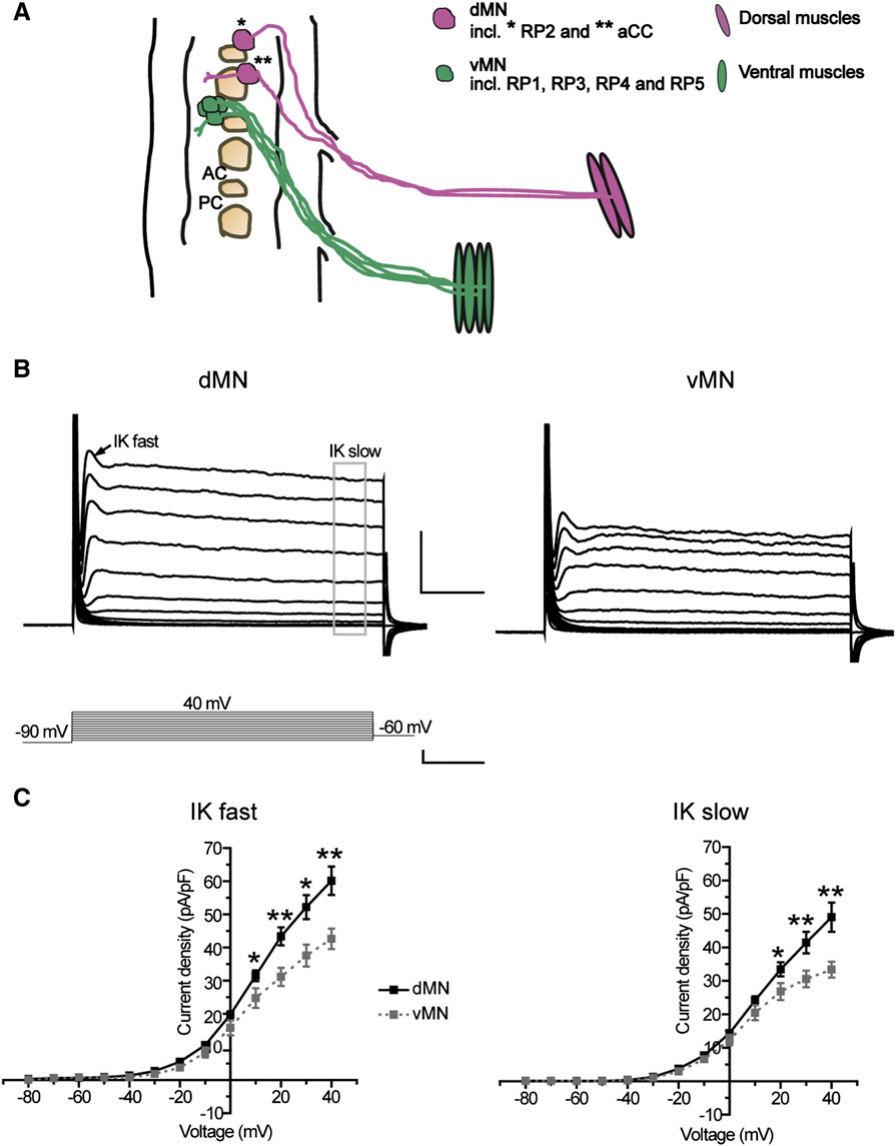
Motoneurons Have Subtype-Specific K^+^ Current Profiles (A) Schematic representation of dorsal and ventral motoneurons (dMNs and vMN, respectively) within the ventral nerve cord of young first-instar larvae and their muscle targets in one half segment. dMNs (magenta) comprise the two Eve positive motoneurons aCC (^∗^) and RP2 (^∗∗^), that project to dorsal muscles (magenta). vMNs (green) comprise the Islet positive motoneurons RP1, RP3, RP4, and RP5 (not individually indicated), that project to ventral muscles (green). AC, anterior commissure; PC, posterior commissure. (B) Average total K^+^ current recorded from dMNs and vMNs are shown. Currents shown are the composite averages made by combining currents obtained from at least eight individual neurons that were normalized for cell capacitance. The voltage-clamp protocol (bottom trace) was −90 mV for 100 msecs prior to voltage jumps of Δ10 mV increments/50 ms duration. Two parameters are measured from the current traces: I_Kfast_ (arrow) was measured at the beginning of the response and I_Kslow_ (gray box) was measured at the end of the voltage step. Scale bars 20 pA/pF and 10 ms for currents and 50 mV/10 ms for the voltage clamp protocol. (C) Current-voltage (IV) plots show significant differences in magnitude of I_Kfast_ and I_Kslow_ in the two motoneuron populations. Both I_Kfast_ and I_Kslow_ are larger in dMNs (black lines) compared to vMNs (gray lines). Values shown are means ± SEM (n ≥ 8).

A crucial test of the hypothesis that Islet regulates ion channel gene expression is the demonstration that membrane electrical properties of Islet-expressing vMNs differ to those of Eve-expressing dMNs. To determine if this is true, we recorded total K^+^ currents from both motoneuron subtypes in first-instar larvae (1–4 hr after hatching; see Figure 1A). Motoneurons were initially identified on the basis of their medial dorsal position in the ventral nerve cord; following electrophysiological patch clamp recordings precise subtype was confirmed on the basis of axonal projection that was visualized by dye filling. We did not observe differences within either subgroups; therefore, recordings have been pooled for the vMN or dMN subtypes.

Figure 1B shows averaged total outward K^+^ currents recorded from both the dMNs and vMNs. The outward K^+^ current is composed of a fast-activating and inactivating component, (I_Kfast_, indicated by the arrow in Figure 1B) and a slower-activating, noninactivating component, (I_Kslow_, indicated by the box in Figure 1B). Analyzing current densities for I_Kfast_ and I_Kslow_ (Figure 1C) shows that dMNs have significantly larger outward K^+^ currents compared to vMNs (Figure 1C; at holding potential of +40 mV I_Kfast_: 60.1 ± 4.3 versus 42.6 ± 3.1 pA/pF; I_Kslow_: 49.0 ± 4.4 versus 33.3 ± 2.4 pA/pF, dMNs versus vMNs, respectively, p ≤ 0.01). Thus, vMNs and dMNs differ in their electrical properties.

The CNS of a first-instar larva is a mature functional neural network in which synaptic transmission is active. Hence, the differences we observe in K^+^ currents could be established entirely due to network activity. Alternatively, subtype specificity might be determined prior to neuronal network formation and, as such, could be considered an intrinsic property of the specific motoneurons. To determine this experimentally, we repeated our analysis following complete block of synaptic transmission (i.e., absence of network activity), achieved through expressing tetanus toxin light chain (TeTxLC) throughout the entire CNS. Using the GAL4^1407^ driver, TeTxLC was expressed pan-neuronally starting at the early neuroblast stage. Since TeTxLC-expressing embryos do not hatch, we recorded K^+^ currents just prior to expected hatching (at late embryonic stage 17). At this stage motoneurons have become fully functional components of the motor network (Baines and Bate, 1998). We found that I_K_ was not significantly perturbed, in either dMNs or vMNs, by blockade of synaptic release. Moreover the difference in K^+^ currents between the dMNs and vMNs was maintained for both I_Kfast_ and I_Kslow_. That differences in I_K_ levels between dMNs and vMNs are established and maintained in the absence of synaptic release strongly suggests that they arise from intrinsic developmental mechanisms independent of evoked synaptic transmission.

### Islet Determines the Electrical Properties of Ventrally Projecting Motoneurons

**Figure 2:**
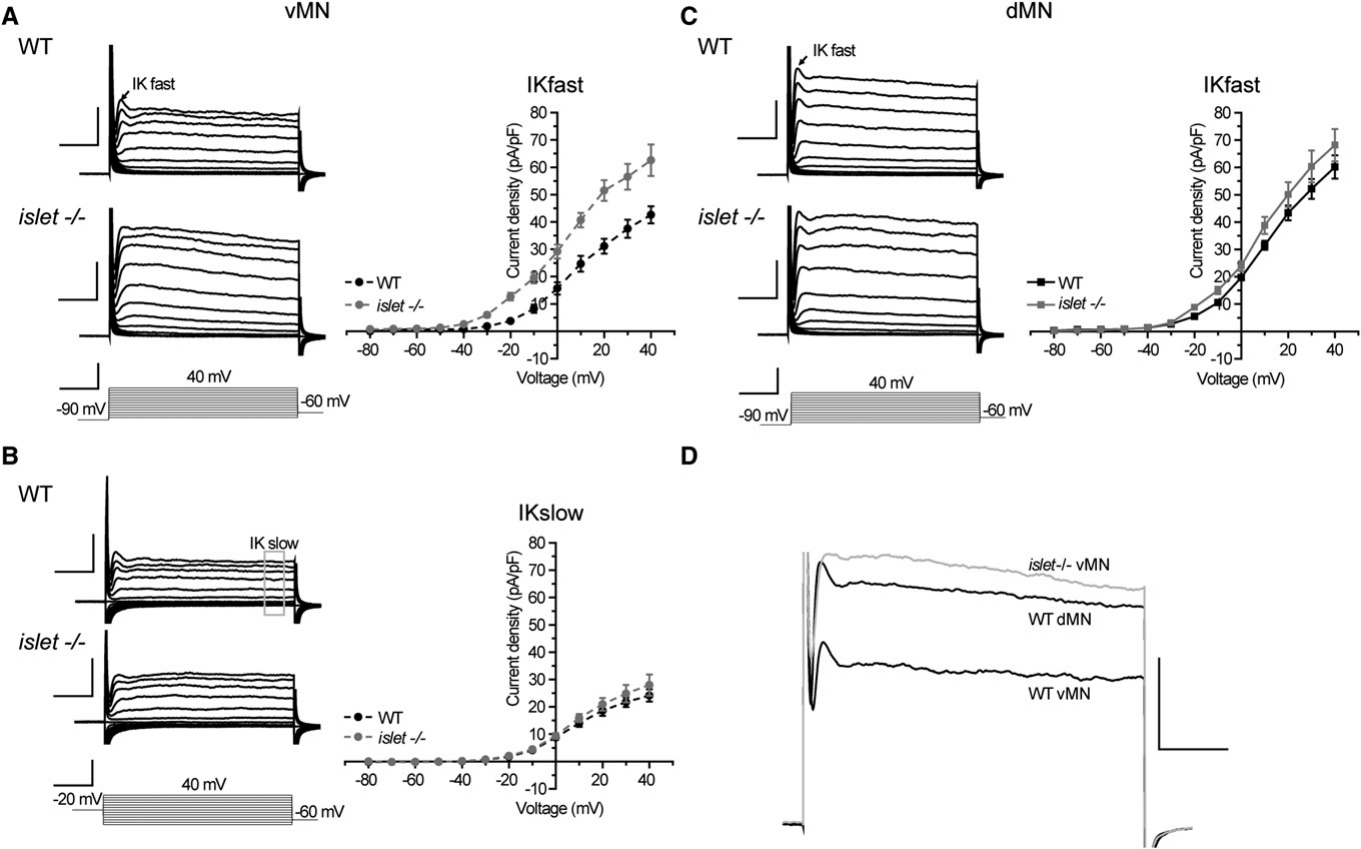
Islet Regulates K^+^ Currents in Ventral, but Not Dorsal, Motoneurons (A) Shows composite averaged K^+^ currents (representing the average from at least eight individual neurons) and respective IV plots for WT and *islet*^*−/−*^ mutant vMNs. Voltage-clamp protocol as in Figure 1. Current density of I_Kfast_ of vMNs (obtained from a prepulse of −90mV) is significantly larger in *islet*^*−/−*^ compared to WT at all test potentials above −40 mV. (B) Neurons were subjected to a prepulse of −20 mV to inactivate I_Kfast_. The remaining I_Kslow_ of vMNs is indistinguishable between *islet*^*−/−*^ and WT. (C) Measurement of I_Kfast_ (obtained from a prepulse of −90mV) in dMNs in *islet*^*−/−*^ and WT are not different. Values shown are means ± SEM (n ≥ 8). (D) Averaged responses of WT dMNs, WT vMNs, and *islet*^*−/−*^ vMNs evoked by the highest test potential (−90 mV prepulse and +40 mV test) are superimposed. The absence of *islet* from vMNs increases K^+^ current magnitude to WT dMNs levels. Scale bars are 20 pA/pF and 10 ms for voltage-clamp responses and 100 mV/10 ms for voltage-clamp protocol.

*Drosophila* larval motoneurons that project axons to ventral muscles express Islet, while those that innervate dorsal muscles express Eve. Loss of *islet* is sufficient to direct ventral-projecting axons dorsally and loss of *eve* to direct dorsal-projecting axons ventrally (Landgraf et al., 1999; Thor and Thomas, 1997). These two distinct motoneuron subtypes provide, therefore, a tractable system to test whether the differences we observe in K^+^ conductance is also intrinsically determined. In order to test whether Islet is able to influence K^+^ currents we recorded from vMNs in an *islet* null (−/−) mutant. This analysis indicated that Islet is sufficient to regulate K^+^ conductance in these motoneurons. Thus, peak current density for I_Kfast_ was significantly increased in homozygous *islet*^−/−^ mutants (Figure 2A; WT 42.6 ± 3.1 versus *islet* 62.6 ± 5.8 pA/pF p ≤ 0.05). By contrast, I_Kfast_ in heterozygous siblings (+/−) did not differ from WT (data not shown). To better measure I_Kslow_ we inactivated I_Kfast_ by applying a −20mV prepulse (100 ms; see Baines and Bate, 1998). Figure 2B shows that loss of *islet* had no effect on I_Kslow_ (WT 24.2 ± 2.3 versus *islet* 28 ± 3.9 pA/pF p = 0.45). We also compared voltage-gated inward currents (i.e., I_Na_ and I_Ca_) in vMNs of heterozygous *islet*^+/−^ and homozygous *islet*^*−/−*^ mutants. Loss of *islet* did not affect the peak current densities of either current (I_Na_: −23.4 ± 2.7 versus −19.7 ± 2.4 and I_Ca_: −19.69 ± 1.68 versus −21.03 ± 2.43, *islet*^*+/−*^ versus *islet*^*−/−*^). Thus, loss of *islet* results in a selective increase in only I_Kfast_ in the vMNs.

To test for autonomy of effect, we also recorded from dMNs in the *islet*^*−/−*^ mutant. Dorsal MNs do not express *islet*, and I_Kfast_ currents of WT and mutant larvae were statistically indistinguishable (Figure 2C; WT 60.1 ± 4.3 pA/pF versus *islet*^*−/−*^ 68.2 ± 5.9 pA/pF p = 0.28). We conclude that loss of *islet* only affects I_Kfast_ in vMNs in which it is normally expressed, but not in dMNs that lack expression of this transcription factor. We further noted that loss of *islet* from the vMNs resulted in a transformation of I_Kfast_ to recapitulate the magnitude of this same current recorded in dMNs. When averaged responses of *islet*^*−/−*^ vMNs and WT dMNs were superimposed, only small kinetic differences remain (Figure 2D). Such an observation is entirely consistent with, and indeed predictive of, the magnitude of I_Kfast_ being regulated by endogenous expression of Islet.

### Islet Represses a DTx-Sensitive Current

Fast K^+^ currents in *Drosophila* neurons are encoded by one or more of at least three different genes: two voltage-gated fast-activating and inactivating channels (A-currents) termed *Shal* and *Shaker* (*Sh*) and a Ca^2+^-activated BK channel termed *slowpoke* (Baker and Salkoff, 1990; Elkins et al., 1986; Singh and Wu, 1990). To determine which K^+^ current is increased in vMNs following loss of *islet*, we used specific blockers of these individual currents. We first explored whether I_Kslowpoke_ is repressed by Islet. To do so we added Cd^2+^ to the bath solution. Cd^2+^ blocks Ca^2+^ entry and, as a consequence, prevents activation of Ca^2+^-activated K^+^ channels. Addition of Cd^2+^ did not diminish the increase in I_Kfast_ observed in the vMNs in *islet*^*−/−*^ mutants (data not shown). We conclude from this that Islet does not influence I_Kslowpoke_.

**Figure 3:**
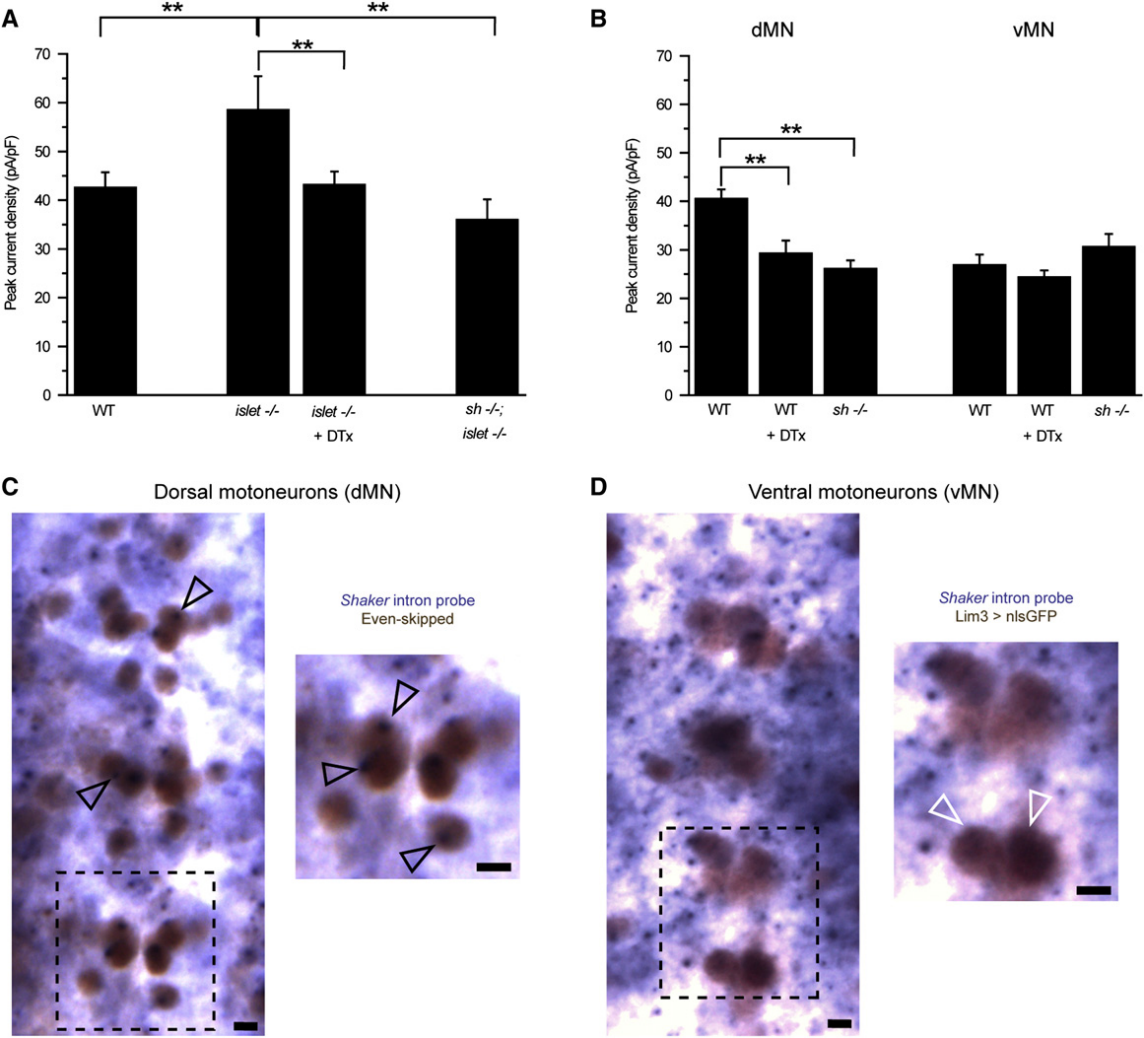
Islet Expression in Ventral Motoneurons Represses a Sh-Mediated K^+^ Current (A) The increase in I_Kfast_ observed in vMNs in the *islet*^*−/−*^ mutant is effectively blocked by the presence of 200 nM DTx in the bath saline indicative that the increased K^+^ current is Sh mediated. This conclusion is further supported by the observation that the effect of removing *islet* on I_Kfast_ requires the presence of *Sh*; no increase is seen in a *Sh*;*islet* double mutant. (B) The presence of DTx significantly reduces I_Kfast_ in dMNs indicative that this neuron subgroup expresses an endogenous Sh-mediated K^+^ current. This is confirmed by a similar reduction in I_Kfast_ observed in a *Sh* null mutant (*Sh*^*−/−*^). By contrast, I_Kfast_ is unaffected in WT vMNs either by exposure to DTx or loss of *Sh*. All recordings are done in the presence of Cd^2+^. Values shown are means ± SEM (n ≥ 8). (C and D) In situ hybridization with *Sh* intron probes. Intron probes detect pre-mRNA at the site of transcription within the nucleus, but not fully processed mRNA in the cytoplasm. The black arrowheads (C) indicate staining for *Sh* transcript in dMN nuclei, labeled with anti-Eve. White arrowheads (D) indicate vMN nuclei, labeled with nuclear GFP, which do not express *Sh*. Early stage 17 embryos were analyzed. Scale bar is 5μm.

By contrast, the presence of α-Dentrotoxin (DTx), a potent and specific blocker for Sh-mediated K^+^ currents (Ryglewski and Duch, 2009; Wu et al., 1989), completely abolishes the increase of I_Kfast_ seen in the vMNs in *islet*^*−/−*^ (Figure 3A; control 58.5 ± 6.9 versus DTx 43.1 ± 2.7 pA/pF p ≤ 0.05). Indeed, I_Kfast_ values obtained in the presence of DTx closely mirrored untreated WT vMNs (43.1 ± 2.7 versus 42.6 ± 3.1 p = 0.9). That DTx negates the *islet*^*−/−*^ phenotype is consistent with Islet inhibiting a Sh-mediated K^+^ current in WT vMNs. To verify this prediction, we recorded I_Kfast_ in a *Sh*;*islet* double mutant. Similarly, under these conditions, peak current density of I_Kfast_ in the double mutant was indistinguishable from WT vMNs (Figure 3A; p = 0.24).

### Sh Is Differentially Expressed in dMNs versus vMNs

Our data are consistent with Islet acting to repress expression of *Sh* in vMNs. Moreover, removal of this repression results in expression of Sh-mediated K^+^ channels that confer “dorsal-like” electrical properties. This model posits, therefore, that dMNs normally express a Sh-mediated K^+^ current.

To test this, we compared I_Kfast_ in dMNs between WT and in the presence of either DTx or in a *Sh* null mutant (*Sh*[14]). We performed these recordings in the presence of external Cd^2+^ to block Ca^2+^-activated fast K^+^ currents. Both acute block of Sh activity (DTx) and loss of function of *Sh* expression significantly reduced I_Kfast_ (Figure 3B; WT 40.5 ± 1.9 versus WT + DTx 29.3 ± 2.7 versus *Sh*[14] 26.1 ± 1.7 pA/pF; p ≤ 0.01 and p ≤ 0.01, respectively). Moreover, the I_Kfast_ recorded in dMNs under both conditions (WT + DTx 29.3 ± 2.7 and *Sh*[14] 26.1 ± 1.7 pA/pF) was indistinguishable from that of vMNs in WT (26.1 ± 2.3 pA/pF, DTx p = 0.38, *Sh* p = 1), which is in full agreement with our model. To further support the notion that the difference in I_Kfast_ that exists between dMNs and vMNs is due, at least in part, to expression of *Sh* in dMNs, we recorded I_Kfast_ in vMNs under the same conditions. As expected, neither the presence of DTx, nor loss of *Sh*, had any marked effect on I_Kfast_ in vMNs (p = 0.51 and 0.23, respectively; Figure 3B).

To further verify the differential expression of *Sh* in dMNs versus vMNs we assessed transcription of *Sh* in these two cell types by in situ hybridization. We designed probes that specifically recognize the *Sh* pre-mRNA. These intron probes label the unspliced *Sh* transcript at the site of transcription within the nucleus, but not the fully mature message in the cytoplasm. We detected *Sh* transcription in dMNs, labeled with Eve antibody (Figure 3C, black arrowheads), but not in vMNs, labeled by expression of GFP (Lim3 > nlsGFP; Figure 3D, white arrowheads). Taken together, both electrophysiology and in situ hybridization are consistent with dMNs expressing *Sh* while the vMNs do not.

### Islet Is Both Necessary and Sufficient to Repress Sh-Mediated K^+^ Currents

**Figure 4:**
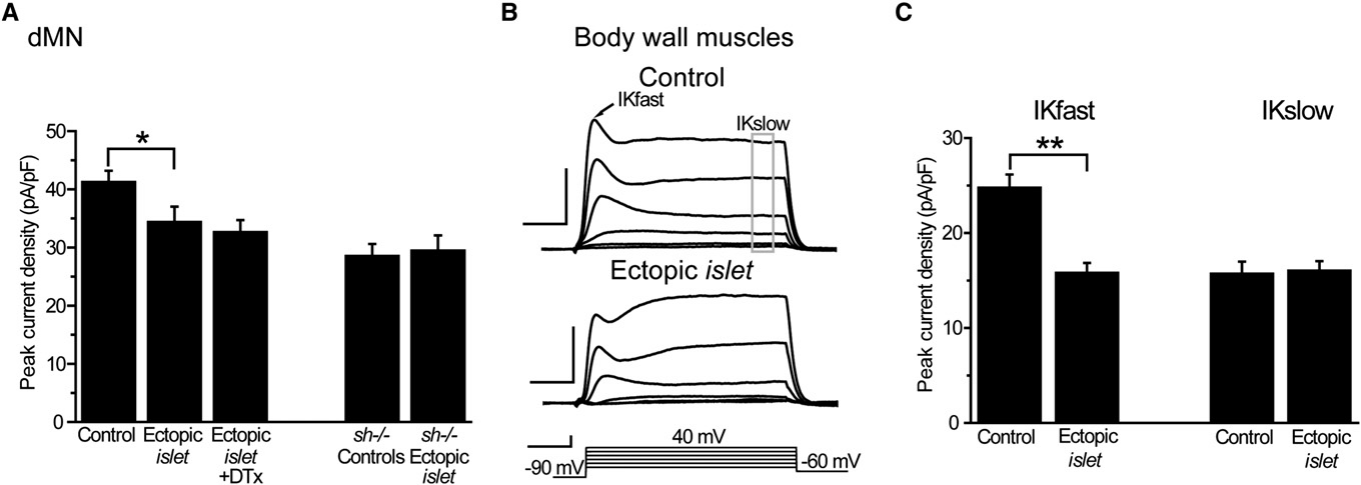
Ectopic Expression of *islet* Is Sufficient to Reduce a Sh-Mediated K^+^ Current (A) Selective expression of *islet* in dMNs is sufficient to significantly decrease I_Kfast_ compared to controls (average of WT and heterozygous GAL4^1407^). Simultaneous application of DTx did not further reduce I_Kfast_. Ectopic *islet* expression also had no effect on I_Kfast_ in a *Sh*^*−/−*^ mutant. Taken together, this data is indicative that *islet* decreases a Sh-mediated K^+^ current in dMNs. Recordings were carried out in the presence of external Cd^2+^ to block Ca^2+^-activated K^+^ currents. (B) Expression of *islet* in body wall muscle results in a significant reduction in I_Kfast_. In low external Ca^2+^ (0.1 mM) I_Kfast_ in these muscles is mediated solely by Sh (see text for details). Traces show averaged composite K^+^ currents, obtained from at least eight individual muscle 6 recordings, in control and *islet* overexpression background. The prominent I_Kfast_ (arrow) of control muscles is significantly reduced when *islet* is ectopically expressed. Scale bar 10 pA/pF 10 ms for current and 50 mV/10 ms for voltage protocol. (C) Averaged peak current densities of I_Kfast_ and I_Kslow_ are shown. Ectopic expression of *islet* significantly reduces I_Kfast_ but has no effect on I_Kslow_. Values shown are means ± SEM (n ≥ 8).

Next, we tested whether Islet is sufficient to repress Sh-mediated K^+^ currents in cells where *Sh*, but not *islet*, is normally expressed. We used two different preparations for these experiments. First, we ectopically expressed *islet* in dMNs. Driving a UAS-*islet* transgene with GAL4^RN2-0^ significantly reduced I_Kfast_ (34.4 ± 2.6 versus 41.2 ± 1.9 pA/pF, experimental versus controls which consisted of WT and heterozygous GAL4 driver line, p ≤ 0.05; Figure 4A). These recordings were carried out in the presence of external Cd^2+^ to eliminate Ca^2+^-dependent K^+^ currents. The observed reduction in I_Kfast_ in dMNs could, however, be due to a reduction in either Sh- or Shal-mediated K^+^ currents. To distinguish between these two possibilities, we tested for DTx sensitivity, which is observed in WT dMNs and is an indicator for the presence of Sh currents. DTx sensitivity was lost when *islet* was ectopically expressed in dMNs (Figure 4A). In addition, when we expressed ectopic *islet* in dMNs in a *Sh*^*−/−*^ background, there was no further reduction in I_Kfast_ compared to ectopic *islet* expression in a WT background (Figure 4A). We conclude from this that ectopic expression of *islet* in dMNs is sufficient to downregulate Sh-mediated I_Kfast_.

The second preparation we used takes advantage of the fact that I_Kfast_ in body wall muscle is solely due to Sh and Slowpoke (the latter of which can be easily blocked [Singh and Wu, 1990]). We recorded from muscle 6 in abdominal segments 3 and 4 in first-instar larvae. To remove the I_Kslowpoke_ component and hence isolate the Sh-mediated I_Kfast_, recordings were done in low calcium (0.1 mM) external saline. Figure 4B depicts the averaged responses from voltage-clamp recordings in control muscle (heterozygous GAL4^24B^ driver, upper trace) and muscle expressing *islet* (lower trace). Peak current densities of I_Kfast_ (entirely due to Sh-mediated K^+^ current) and the slow noninactivating currents recorded at +40 mV are shown in Figure 4C. Ectopic expression of *islet* in muscle is sufficient to produce a significant reduction in I_Kfast_ (control 26.6 ± 2.4 versus 24B > *islet* 15.8 ± 1.0 pA/pF, p ≤ 0.01) while no effect was seen on the slow current. Thus, expression of *islet* in dMNs is sufficient to reduce a DTx-sensitive component of I_Kfast_. Similar expression in muscle clearly demonstrates that Islet is sufficient to downregulate a Sh-mediated fast K^+^ current.

### Islet Binds Directly to the Sh Locus

**Figure 5:**
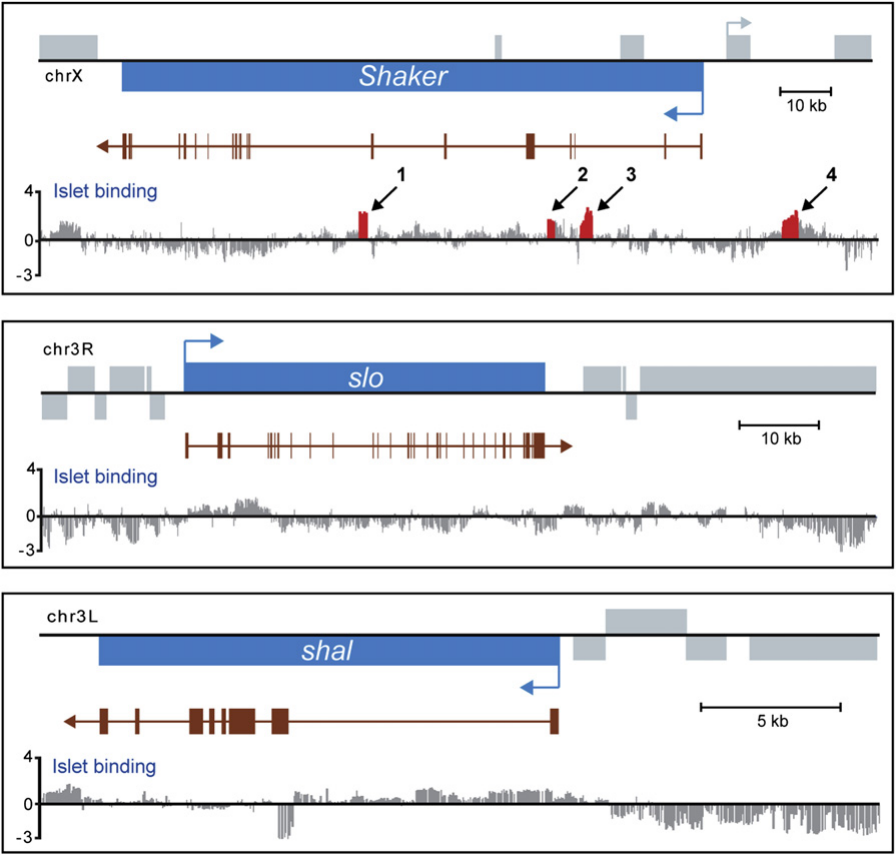
DamID Demonstrates Direct Binding of Islet to the *Sh* Locus In Vivo Of the genes encoding the three known fast K^+^ current channels in *Drosophila,* Islet binds to *Sh*, but not *Slo* or *Shal*. The transcription units of *Sh*, *Slo*, and *Shal* are shown in blue with blue arrows indicating the direction of transcription. Grey vertical bars indicate the position of oligonucleotide probes on the genomic microarray. Bar heights show the average of normalized log2-transformed ratios from 3 independent DamID experiments with those in red, and indicated with arrows, showing a significant peak within the data set (FDR < 0.1%). The *Sh*, *Slo*, and *Shal* transcripts are shown in brown with vertical bars/boxes representing exons. Additional transcription units within the region are shown as gray boxes.

Our electrophysiology indicates that Islet is able to repress Sh-mediated K^+^ current. To identify putative targets of Islet we used DamID, a well-accepted technique for demonstrating direct binding to chromatin or DNA in vivo (Choksi et al., 2006; Filion et al., 2010; Southall and Brand, 2009; van Steensel and Henikoff, 2000). Our analysis identifies 1,769 genes (exhibiting one or more peaks of Islet binding within 5 kb of the transcriptional unit) as direct targets of Islet (FDR < 0.1%). Consistent with our model of Islet regulating a Sh-mediated K^+^ current, we find three significant binding sites within introns of the *Sh* locus (arrows 1 to 3 in Figure 5). Intragenic binding of transcription factors is common in both vertebrates (Robertson et al., 2007) and invertebrates (Southall and Brand, 2009). A fourth significant peak is found upstream of *Sh* (arrow 4 in Figure 5). Binding of Islet at this site could regulate the expression of either *Sh* and/or *CG15373* an adjacent, divergently transcribed, gene. By contrast, *Shal* and *slowpoke*, which also encode fast neuronal K^+^ currents, were not identified as putative targets (Figure 5). Thus, these data show that Islet binds to the *Sh* locus and is likely to regulate transcription of the *Sh* gene directly.

To confirm that Islet binds *Sh* and regulates its transcription, we used qRT-PCR to quantify levels of *Sh* transcripts. We compared *Sh* transcript levels in larval CNS between control, *islet*^*−/−*^ and panneuronal *islet* expression (1407 > *islet*). In comparison to control, the absence of *islet*^*−/−*^ resulted in a 27% increase in *Sh* (1.27 ± 0.01, n = 2, p < 0.05). By contrast, panneuronal expression of transgenic *islet* resulted in a 45% decrease in *Sh* transcript (0.45 ± 0.06, n = 2, p < 0.05). We also measured *Sh* transcript level in body wall muscle following ectopic expression of *islet* (24B > *islet*). Similar to the CNS, *Sh* transcripts were reduced by 31% relative to control (0.31 ± 0.01, n = 2, p < 0.05). Taken together with the results obtained by DamID, this strongly suggests that Islet binds to, and represses transcription of, the *Sh* gene.

### Sh Regulates Action Potential Frequency

**Figure 6:**
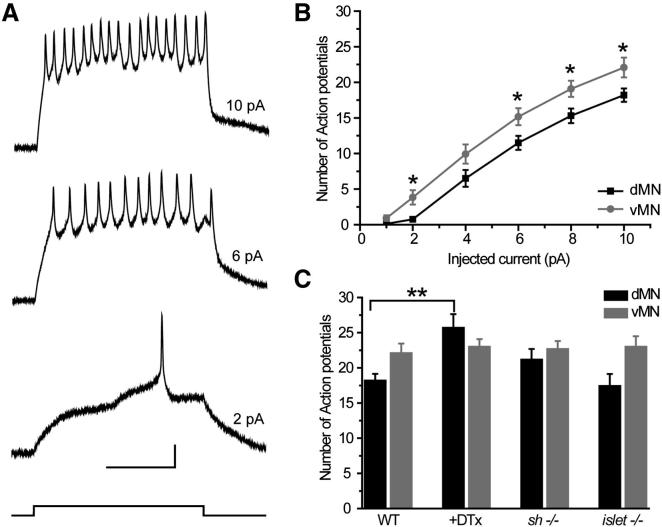
Membrane Excitability Differs between dMNs and vMNs (A) Example of a whole cell current clamp recordings obtained from a dMN (aCC). Responses to 500 ms depolarizing current pulses of 2, 6, and 10 pA are shown. An example of a current step is shown underneath the responses. Scale bar is 10 mV/200 ms. (B) Number of action potentials fired per 500 ms current step by dMNs and vMNs are plotted against the amplitude of injected current. dMNs fire significantly less action potentials than vMNs at most current steps. (C) Number of action potentials evoked by a 10 pA current injection. WT dMNs fire significantly less action potentials than vMNs. Removal of Sh-dependent K^+^ current by DTx or *Sh*^*−/−*^ increases action potential firing in dMNs to levels seen in vMNs. Action potential firing in vMNs remains unaffected. Removal of *islet* (*islet*^*−/−*^) also has no effect on firing in either dMns or vMNs. Values shown are means ± SEM (n ≥ 8).

Voltage-dependent K^+^ currents, such as those mediated by Sh, contribute to setting membrane excitability (and thus the ability to fire action potentials) (Goldberg et al., 2008; Peng and Wu, 2007). These currents are therefore critical for network function and the generation of appropriate behaviors (Smart et al., 1998). It has been shown that modulation of Sh-mediated current, using dominant-negative transgenes, can bring about significant changes in excitability (Mosca et al., 2005). We were interested in whether and how excitability differs between motoneurons that express a Sh-mediated K^+^ current (dMNs) and those that do not (vMNs). We recorded excitability in current clamp. Typical responses are shown in Figure 6A. We found that dMNs fired significantly fewer action potentials than vMNs at most current steps (Figure 6B; 10 pA: 18.2 ± 0.9 versus 22.1 ± 1.4 p = 0.04; 8 pA: 15.3 ± 1.0 versus 19.1 ± 1.1 p = 0.02; 6 pA: 11.5 ± 1.0 versus 15.2 ± 1.2 p = 0.04; 4 pA: 6.5 ± 1.2 versus 9.9 ± 1.4 p = 0.09; 2 pA: 0.8 ± 0.3 versus 3.8 ± 1.0 p = 0.03; 1 pA: 0.1 ± 0.1, versus 0.9 ± 0.4: p = 0.13; dorsal versus ventral, respectively). The above results suggest that the Sh-mediated K^+^ current (expressed only in dMNs) reduces action potential (APs) firing when present.

To validate this conclusion, we reduced Sh current in dMNs acutely by adding DTx to the bath and recorded AP firing. AP firing increased from 18.2 ± 0.9 APs (WT) to 25.7 ± 1.9 APs (DTx, p < 0.05; Figure 6C). A similar result, although not significant, was obtained when APs were recorded from dMNs in a *Sh* mutant (18.2 ± 0.9 to 21.2 ± 1.5 APs, p = 0.07; Figure 6C). Indeed, in both treatments, firing rates between dMNs and vMNs were indistinguishable (*Sh*^−/−^ 21.2 ± 1.5 versus 22.7 ± 1.1; DTx 25.7 ± 1.9 versus 23.0 ± 1.8 APs, dMNs versus vMNs respectively, p > 0.05; Figure 6C). As predicted, vMN excitability was not affected by either DTx or loss of *Sh* (22.1 ± 1.4 versus 23.0 ± 1.8 versus 22.7 ± 1.1, WT, DTX, *Sh*^*−/−*^, respectively, p > 0.05; Figure 6C). Perhaps unexpectedly, the increase in I_Kfast_ in vMNs, which results from the loss of *islet*, did not influence AP firing. Loss of *islet* also had no effect on APs fired in dMNs which is predictable because dMNs do not express this protein (Figure 6C). Finally, determination of AP firing in a *Sh*;*islet* double loss of function mutant revealed no additional effects: AP firing is increased in dMNs and unaffected in vMNs (data not shown). Why loss of *islet*, which increases I_Kfast_ in vMNs, does not influence AP firing in these neurons is unknown, but may be indicative of additional homeostatic mechanisms.

## Discussion

Diversity in neuronal electrical properties is dictated by the type, location, and number of ion channels expressed in individual neurons. While activity-dependent mechanisms that act to adjust these properties in mature neurons have been studied in detail (Davis and Bezprozvanny, 2001; Spitzer et al., 2002), the mechanisms that specify electrical properties in embryonic neurons, prior to network formation, are not understood. These mechanisms are, however, likely to be part of cell-intrinsic programs of specification. The demonstration of differential expression of transcription factors between neuronal cell types underpins the proposal of a combinatorial code sufficient to determine key aspects of neuron specification, including axon guidance and neurotransmitter phenotype (Polleux et al., 2007; Shirasaki and Pfaff, 2002; Thor and Thomas, 2002). However, whether these same factors are sufficient to set cell-specific electrical characteristics remains unknown.

A wealth of studies on motoneuron specification, from flies to mammals, has shown that early developmental decisions, such as subclass identity, is dictated, at least in part, by a code of transcription factors (Dasen et al., 2005, 2008; De Marco Garcia and Jessell, 2008; Landgraf et al., 1999; Landgraf and Thor, 2006; Thor and Thomas, 1997). With its relatively simple CNS and powerful molecular genetics, *Drosophila* has been central to these studies. Embryonic *Drosophila* motoneurons express a stereotypic mix of identified transcription factors which are evolutionary conserved with mammals (Thaler et al., 1999, 2002; Thor and Thomas, 1997). Motoneurons which predominantly innervate ventral muscles express *islet*, *Lim3*, and *dHb9*. Motoneurons which project dorsally express *eve* (Landgraf et al., 1999; Landgraf and Thor, 2006; Thor and Thomas, 1997). A first indication that ion channel genes may also be targets of these transcription factors was provided by our demonstration that overexpression of *eve* was sufficient to alter the outward voltage-gated K^+^ current through transcriptional repression of *slowpoke* (encoding a BK Ca^2+^-activated K^+^ channel) in *Drosophila* motoneurons (Pym et al., 2006). However, while a common developmental regulation of neuronal morphology and function, at least in motoneurons, might be inferred from this study, only Eve-positive cells were investigated. This leaves open the question, whether Eve, or for that matter any of the other transcription factors, is deterministic for specific membrane currents.

The principle of duality in role for transcription factors such as Eve and Islet is significant because it is predictive that neuron morphology and electrical signaling are, at least in part, determined by common developmental mechanisms. Studies of vertebrate homologs of these transcription factors, widespread in the mammalian CNS, provide additional support for such a scenario. For example, Islet-1 and Islet-2 are known to regulate neuron identity, axonal guidance and choice of neurotransmitter in vertebrate CNS (Hutchinson and Eisen, 2006; Segawa et al., 2001; Thaler et al., 2004). Associated microarray analysis on murine mutant tissue identifies ion channels as putative targets of Islet-1, including Shal-related K^+^ channel *Kcnd2* and Na^+^ channel *Na*_*v*_*1.8*. Regulation of expression has, however, yet to be demonstrated (Sun et al., 2008). It is conceivable that in zebrafish recently reported differences in outward K^+^ currents between two embryonic motoneurons, dorsal MiP and ventral CaP (Moreno and Ribera, 2009), may be regulated by the differential expression of Islet1/2 in these neurons (Appel et al., 1995).

We provide substantial evidence that differential expression of *islet* in vMNs versus dMNs is critical for determining subtype-specific differences in Sh-mediated K^+^ currents. Because these Sh-mediated K^+^ currents regulate action potential frequency, they will contribute to network function. Comparable to our findings in *Drosophila*, in both the mouse cochlea and cortex, neurons that fire only a small number of action potentials to a given current pulse (termed rapidly adapting) express a DTx-sensitive Kv1 (Sh-like) K^+^ current. By contrast, neurons that fire many action potentials (slowly adapting) do not. The firing pattern of rapidly adapting neurons can be transformed into that of slowly adapting neurons by application of the Sh-specific blocker DTx (Miller et al., 2008). Our own data are consistent with such a role for Sh because we show that dMNs which express *Sh*, fire fewer action potentials than vMNs. Moreover, the number of action potentials fired by dMNs is increased by genetic or pharmacological block of the Sh-mediated K^+^ current. We envisage, therefore, that regulation of action potential firing, through Islet-mediated transcriptional control of a Sh-like K^+^ current, might be well conserved.

While the presence of early factors able to regulate ion-channel gene expression is predictive of predetermination of electrical signaling properties in embryonic neurons, a challenge remains to understand how individual neurons decode this information. In the *Drosophila* ventral nerve cord, we find that the presence or absence of a Sh-mediated K^+^ current is determined by whether *islet* is expressed or not. Thus, Islet seems to act as a binary switch; when present it prevents expression of *Sh* and vice versa. However, it seems unlikely that all combinatorial factors act in this way. For example, the activity of Eve seems to be related to its relative level of expression, since endogenous Eve only partially represses transcription of *slowpoke* (a Ca^2+^-dependent K^+^ channel) in the dorsal motoneuron aCC (Pym et al., 2006). It remains to be determined whether efficacy of regulatory activity is specific to individual transcription factors or to target genes.

We show here that the Lim-homeodomain transcription factor Islet forms part of an intrinsic “decision-making” process that is critical to specifying subtype-specific electrical properties in developing motoneurons. It might be argued that input from pre- and postsynaptic partners is involved in setting early electrophysiological differences between neurons. Indeed such inputs play a pivotal role during axonogenesis and synapse development. Blocking all synaptic transmission showed that neural network activity is not required to establish early electrophysiological differences between motoneuron subgroups. Motoneurons also receive instructive cues from their postsynaptic muscle targets during NMJ development (Fitzsimonds and Poo, 1998). In this regard it is significant that the difference in I_Kfast_ we observe between dMNs and vMNs is abolished in a myosin heavy chain mutant (*mhc1*) that fails to produce contractile muscles. Indeed, I_Kfast_ is decreased in dMNs to the level seen in WT vMNs (V.W. and R.A.B., unpublished observations). This is, perhaps, indicative that the dMNs require an instructive signal from their muscle targets in order to follow a different path of electrical development. Whether this path suppresses *islet* expression in dMNs remains to be determined. Significantly, vMNs were not affected in the *Mhc1* mutant suggesting that repression of Sh-dependent I_K_ by Islet is independent of muscle derived input.

Why do motoneurons differ in their electrical properties and what is the functional implication? dMNs and vMNs receive differential synaptic drive (Baines et al., 2002) and innervate distinct muscle targets, dorsal obliques and ventral longitudinals, respectively (Landgraf et al., 1997). During larval crawling ventral muscles are recruited prior to dorsal muscles (Fox et al., 2006) to, probably, facilitate coordinated movement. Interestingly, synaptic strength, based on EJP amplitude, is largest between vMNs and their target muscles. While the precise underlying mechanism is unknown, pharmacology suggests that terminals of dMNs express a larger Sh-dependent K^+^ current compared to vMNs. This current disproportionately reduces presynaptic neurotransmitter release and hence regulates synaptic strength (Lee et al., 2008). Whether this alone can account for the delay of dorsal muscle contraction is not known. Differences in electrical properties, specifically delay to first spike, have also been observed between *Drosophila* motoneurons (Choi et al., 2004). While the precise reasons for these differences remain speculative, they are consistent with differential contribution to muscle activity that underlies locomotion in *Drosophila* larvae.

We can recapitulate the repressive effect of ectopic *islet* expression on Sh-mediated K^+^ current in body wall muscle. This is important for two reasons. First, it provides unequivocal support for the hypothesis that Islet is deterministic for expression of *Sh* in excitable cells, regardless of whether those cells are neurons or muscle. Second, body wall muscles are isopotential and do not therefore suffer from issues of space clamp (Broadie and Bate, 1993). Analysis of ionic currents in neurons can be complicated by such factors, which becomes more serious for analysis of those currents located further away from the cell body in the dendritic arbor. Hence electrophysiological-tractable muscles may offer the possibility to derive a more complete understanding of the differential activity of codes of transcription factors on the regulation of ion channel development within the developing nervous system.

## Experimental Procedures

### Fly Stocks

For larval collections, flies were transferred into laying pots and allowed to lay eggs onto grape juice agar plates. Laying pots were kept at 25°C and 18°C for motoneuron and muscle experiments, respectively. The following fly strains were used: Canton-S as wild-type (WT), *islet* mutant tup[isl-1] rdo[1] hk[1] pr[1]/Cyo act::GFP (rebalanced from Bloomington 3556), *Shaker* mutant Sh[14] (Bloomington 3563, carries the KS133 mutation). The *Shaker* and *islet* mutations were combined in a double mutant Sh[14];tup[Isl-1]/CyO act::GFP. The *islet* mutants and *Sh*;*islet* double mutants are embryonic lethal; however, a few homozygous escapers are viable up until the first-instar larval stage. Transgenes were expressed in a tissue-specific manner using the GAL4/UAS system (Brand and Perrimon, 1993). The driver line GAL4^1407^ (homozygous viable on the second chromosome) was used to express UAS containing transgenes carrying the active (UAS-TNT-G) or inactive (UAS-TNT-VF) form of tetanus toxin light chain (TeTxLC) in all CNS neurons (Sweeney et al., 1995). GAL4^Lim3^ was used to express GFP in vMNs for in situ hybridization. GAL4^RN2-0^ (homozygous viable on the second chromosome) or GAL4^RRa^ (homozygous viable on the 3^rd^ chromosome) were used to express *islet* (UAS-*islet* x2) in dMNs. GAL4^24B^ (homozygous viable on the second chromosome) was used to express *islet* (UAS-*islet* x2) body wall muscle. The dMN driver GAL4^RRa^ as well as the UAS-*islet* construct were crossed into the Sh[14] mutant background.

### Embryo and Larval Dissection

Newly hatched larvae or late stage 17 embryos were dissected and central neurons were accessed for electrophysiology as described by Baines and Bate (1998). For muscle recordings newly hatched larvae were dissected as for CNS electrophysiology, but the CNS was removed. The muscles were treated with 1 mg/ml collagenase (Sigma) for 0.5 to 1 min prior to whole cell patch recording. Larvae were visualized using a water immersion lens (total magnification, 600×) combined with DIC optics (BX51W1 microscope; Olympus Optical, Tokyo, Japan).

### Electrophysiology

Recordings were performed at room temperature (20°C to 22°C). Whole-cell recordings (current and voltage clamp) were achieved using borosilicate glass electrodes (GC100TF-10; Harvard Apparatus, Edenbridge, UK), fire-polished to resistances of between 15 - 20 MΩ for neurons and between 5 and 10 MΩ for muscles.

Neurons were identified based on their position within the ventral nerve cord. Neuron type was confirmed after recording by filling with 0.1% Alexa Fluor 488 hydrazyde sodium salt (Invitrogen), which was included in the internal patch saline. Recordings were made using a Multiclamp 700B amplifier controlled by pClamp 10.2 (Molecular Devices, Sunnyvale, CA). Only neurons with an input resistance > 1 GΩ were accepted for analysis. Traces were sampled at 20 kHz and filtered at 2 kHz. The voltage-clamp protocols used to record total K^+^ currents were as follows: for neurons, from the resting potential of −60 mV neurons were hyperpolarized to −90 mV for 100 ms, the voltage was then stepped from −80 mV to +40 mV in increments of Δ10 mV for 50 ms. To isolate slow K^+^ currents a prepulse of −20mV for 100 ms was used (Baines and Bate, 1998). For muscles a maintained holding potential of −60 mV was used and a −90 mV prepulse for 200 ms and voltage jumps of Δ20 mV increments were applied from −40 to +40 mV. Leak currents were subtracted off-line for central neuron recordings. For muscle recordings, however, on line leak subtraction (P/4) was used. Recordings were done in at least four animals and at least eight neurons/muscles were recorded from in total for each experiment. Individual recordings were averaged, following normalization relative to cell capacitance, to produce one composite average representative of that group of recordings. Cell capacitance was determined by integrating the area under the capacity transients evoked by stepping from −60 to −90 mV (checked before and after recordings).

Membrane excitability (i.e., action potential firing) was determined using injection of depolarizing current (1, 2, 4, 6, 8, 10 pA/500 ms) from a maintained membrane potential (V_m_) of −60 mV. V_m_ was maintained at −60 mV by injection of a small amount of hyperpolarizing current.

### Solutions

#### Motoneuron Recordings

External saline for dissection and current clamp analysis of excitability consisted of the following (in mM): 135 NaCl, 5 KCl, 4 MgCl_2_·6H_2_O, 2 CaCl_2_·2H_2_O, 5 N-Tris [hydroxymethyl]methyl-2-aminoethanesulfonic acid (TES), 36 sucrose, pH 7.15. For isolation of total K^+^ currents 1 μM TTX (Alomone Labs, Jerusalem, Israel) was added to the external solution. For most recordings Ca^2+^-activated K^+^ currents were eliminated by adding Cd^2+^ (0.2 mM) to the saline. Sh-mediated K^+^ current was blocked using dendrotoxin (DTx, Sigma, 200 nM). Current clamp recordings were done in the presence of mecamylamine (1 mM, Sigma) to block endogenous cholinergic synaptic currents. Internal patch solution consisted of (in mM): 140 K^+^ gluconate, 2 MgCl_2_·6H_2_O, 2 EGTA, 5 KCl, and 20 HEPES, pH 7.4.

#### Muscle Recordings

External saline (Stewart et al., 1994) for dissection and voltage-clamp analysis consisted of the following (in mM): 70 NaCl, 5 KCl, 0.1 CaCl_2_, 20 MgCl_2_·6H_2_O, 10 NaHCO_3_, 5 HEPES, 115 sucrose, 5 trehalose (pH 7.2). The calcium concentration was kept low (0.1 mM) to prevent activation of Ca^2+^-dependent K^+^ currents. Internal patch saline was the same as for neurons.

### In Situ Hybridization

In situ hybridization was performed as previously described (Choksi et al., 2006), using a hybridization temperature of 65°C. Five separate probes were generated to target an intron of Sh common to all splice isoforms (second intron of Sh-RB). The probes were equally mixed before use. The primers used to generate the RNA probes are as follows:

Sh_int1_FW (CTTCTTTCTTGGATTGAAGGACAT), Sh_int1_RVT7 (CAGTAATACGACTCACTATTATAATGCAACAAAATTGAAGCAGAT), Sh_int2_FW (TAGGCATCATTGCACTGTCTTATT), Sh_int2_RVT7 (CAGTAATACGACTCACTATTATAGTAGCCACTCTGAGCACTATGG),
Sh_int3_FW (CACTTTGAGAGTCCTGCAGTTTTA), Sh_int3_RVT7 (CAGTAATACGACTCACTATTATTTGGGTCATTTGTCAAACATATC),
Sh_int4_FW (GCCAAAGAAAACGTGTTAAAATCT), Sh_int4_RVT7 (CAGTAATACGACTCACTATTAGTACCAAGTTTGTTTTTGCATCTG),
Sh_int5_FW (AAAGCAATTCAAGGCACTAAAATC), Sh_int5_RVT7 (CAGTAATACGACTCACTATTAGCTATTTGAAACTTTTCGTCGTTT).

Immunohistochemistry was performed after the in situ protocol using an anti-Eve antibody (1:5,000; Frasch et al., 1987) or an anti-GFP antibody (1:2,000; Abcam ab6556) and developed using DAB.

### Real-Time RT-PCR

Muscle tissue and CNS were collected from newly hatched larvae or late stage 17 embryos. Between 100 and 180 animals were dissected for each genotype. Following RNA extraction (QIAGEN RNaesy Micro kit) cDNA was synthesized using the Fermentas Reverse Aid H minus First strand cDNA synthesis kit, according to the manufacturer's protocol. RNA concentration was matched for control and experimental sample prior cDNA synthesis. qPCR was performed on the Roche LightCycler 1.5 (Roche, Lewes, UK) using the Roche LightCycler FastStart DNA Master SYBR Green reaction mix. The thermal profile used was 10 min at 95°C followed by 40 cycles of 10 s at 95°C, followed by 4 s at 59°C, and finally 30 s at 72°C. Results were recorded using the delta delta C_t_ method and are expressed as Fold difference compared to control (*isl*^*−/−*^ compared to *isl*^*+/−*^, 1407 > *islet* to 1407 > GFP, 24 B > *islet* to 24B > GFP). C_t_ values used were the means of duplicate replicates. Experiments were repeated twice. PCR primers (forward and reverse primers in 5′ to 3′ orientation) were as follows: *rp49* CTAAGCTGTCGCACAAATGG and GGAACTTCTTGAATCCGGTG; *Sh* CAACACTTTGAACCCATTCC and CAAAGTACCGTAATCTCCGA.

### DamID Analysis

A pUASTattB-NDam vector was created (to allow integration of the Dam transgene into a specific site) by cloning the Dam-Myc sequence from pNDamMyc (van Steensel and Henikoff, 2000) into the multiple cloning site of pUASTattB (Bischof et al., 2007) using EcoRI and BglII sites. The full-length coding sequence of *islet* was PCR amplified from an embryonic cDNA library and cloned into pUASTattB-NDam using BglII and NotI sites. Transgenic lines were generated by injecting pUASTattB-NDam (control line) and pUASTattB-NDam-*islet* constructs (at 100ng/μl) into ΦX-22A (with phiC31 expressed in the germline and a docking site at 22A) blastoderm embryos (Bischof et al., 2007). Preparation of Dam-methylated DNA from stage 17 embryos was performed as previously described (Pym et al., 2006). The Dam-only and Dam-*islet* samples were labeled and hybridized together on a whole genome 2.1 million feature tiling array, with 50- to 75-mer oligonucleotides spaced at approximately 55 bp intervals (Nimblegen systems). Arrays were scanned and intensities extracted (Nimblegen Systems). Three biological replicates (with one dye-swap) were performed. Log2 ratios of each spot were median normalized.

A peak finding algorithm with false discovery rate (FDR) analysis was developed to identify significant binding sites (PERL script available on request). All peaks spanning 8 or more consecutive probes (>∼900 bp) over a 2-fold ratio change were assigned a FDR value. To assign a FDR value, the frequency of a range of small peak heights (from 0.1 to 1.25 log2 increase) were calculated within a randomized data set (for each chromosome arm) using 20 iterations for each peak size. This was repeated for a range of peak widths (6 to 15 consecutive probes). All of these data were used to model the exponential decay of the FDR with respect to increasing peak height and peak width, therefore enabling extrapolation of FDR values for higher and broader peaks. This analysis was performed independently for each replicate data set. Each peak was assigned the highest FDR value from the 3 replicates. Genes were defined as targets where a binding event (with a FDR < 0.1%) occurred within 5 kb of the transcriptional unit (depending on the proximity of adjacent genes).

### Statistics

Statistical significance was calculated using a nonpaired t test with a confidence interval of p ≤ 0.05 (^∗^) and ≤ 0.01 (^∗∗^). All quantitative data shown are means ± SEM.
